# High ferritin levels have major effects on the morphology of erythrocytes in Alzheimer's disease

**DOI:** 10.3389/fnagi.2013.00088

**Published:** 2013-12-06

**Authors:** Janette Bester, Antoinette V. Buys, Boguslaw Lipinski, Douglas B. Kell, Etheresia Pretorius

**Affiliations:** ^1^Department of Physiology, Faculty of Health Sciences, University of PretoriaArcadia, South Africa; ^2^Microscopy and Microanalysis Unit, University of PretoriaArcadia, South Africa; ^3^Joslin Diabetes Center, Harvard Medical SchoolBoston, MA, USA; ^4^School of Chemistry and The Manchester Institute of Biotechnology, The University of ManchesterLancs, UK

**Keywords:** Alzheimer's disease, erythrocytes, iron, scanning electron microscopy, atomic force microscopy

## Abstract

**Introduction:** Unliganded iron both contributes to the pathology of Alzheimer's disease (AD) and also changes the morphology of erythrocytes (RBCs). We tested the hypothesis that these two facts might be linked, i.e., that the RBCs of AD individuals have a variant morphology, that might have diagnostic or prognostic value.

**Methods:** We included a literature survey of AD and its relationships to the vascular system, followed by a laboratory study. Four different microscopy techniques were used and results statistically compared to analyze trends between high and normal serum ferritin (SF) AD individuals.

**Results:** Light and scanning electron microscopies showed little difference between the morphologies of RBCs taken from healthy individuals and from normal SF AD individuals. By contrast, there were substantial changes in the morphology of RBCs taken from high SF AD individuals. These differences were also observed using confocal microscopy and as a significantly greater membrane stiffness (measured using force-distance curves).

**Conclusion:** We argue that high ferritin levels may contribute to an accelerated pathology in AD. Our findings reinforce the importance of (unliganded) iron in AD, and suggest the possibility both of an early diagnosis and some means of treating or slowing down the progress of this disease.

## Introduction

There is increasing evidence that vascular components, including RBCs and fibrinogen, play a fundamental role in neurological diseases, including Alzheimer's disease (AD) (Kovacic and Fuster, 2012; Diomedi and Misaggi, 2013; Kling et al., 2013). Erythrocytes (RBCs) are highly deformable, and this physical property contributes significantly to assisting blood flow in the microcirculation, where capillaries may be barely larger than the nominal RBC diameter (Mohandas and Gallagher, 2008). According to one view, the abnormalities in RBCs and their flow contribute to AD by obstructing oxygen delivery to brain (Tateishi et al., 2001; Mohanty et al., 2008; Tripathy et al., 2013) that, in turn, causes hypoxia leading to a chronic inflammation (Eltzschig and Carmeliet, 2011; Wyss-Coray and Rogers, 2012). Mohanty and co-workers in 2010 noted that there is a potential link between alterations in the RBC membrane proteome in AD subjects and AD pathology (Mohanty et al., 2010). These authors showed that 15% of RBCs in AD patients were elongated, and that there were alterations in the RBC membrane architecture. They suggested that this might be due to RBC-beta-amyloid interactions and/or changes in the expression of membrane proteins. Fibrinogen, a plasma precursor of fibrin has been found in the brains of AD patients (Choi et al., 2002) and it was shown that fibrin also interacted with beta-amyloid protein (Merkle et al., 1996) in such patients. There is also evidence in AD that perivascular leakage of fibrinogen happens in AD and more importantly that fibrin accelerates neurovascular damage (Paul et al., 2007).

Closely linked to hematological pathology in AD are increased iron levels, that also play an important role in the pathogenesis of the condition (Barnham and Bush, 2008; Smith et al., 2010; Weinberg, 2010). Increased iron levels cause oxidative stress, as they participate in oxygen-dependent free radical formation (Kell, 2009). Free radical stresses, and specifically those from hydroxyl radicals, are important factors in the pathogenesis of AD, as they lead to protein modification and consequently to neuronal damage (Casadesus et al., 2004; Castellani et al., 2012). This free radical stress may also impact on RBCs, and this may cause extensive and accumulative damage to these cells, ultimately compromising their functioning. Optimal oxygen delivery to the brain clearly depends on optimal RBC physiology (Tateishi et al., 2001). Finally, here, increased RBC aggregation and sedimentation, as well as flow abnormalities, have been observed in the blood of patients with degenerative diseases such as atherosclerosis and inflammation, that are also known to be associated with AD (Robinson et al., 1995; Andresdottir et al., 2003; McMahon et al., 2013).

We have shown previously that when iron overload is present in conditions such as diabetes and hereditary hemochromatosis, RBCs are distorted, and form pointed extensions (Lipinski et al., 2012a; Pretorius, 2013; Pretorius and Lipinski, 2013). This change in morphology (and perhaps deformability—see below) may play a fundamental role in the thrombotic and cardiovascular risk typically seen in these and other conditions. In diseases such as thalassemia and hereditary hemochromatosis (Carpenter et al., 2013), literature also suggests the prevalence of cardiac complications (Noori et al., 2012; Maggio et al., 2013) and thrombocytosis with increased risk of stroke (Shariat et al., 2013). Also, we have previously shown that a changed RBC, platelet and fibrin network ultrastructure is present in stroke (Lipinski et al., 2012b; Swanepoel and Pretorius, 2012).

During wound healing and the consequent activation of the coagulation pathway, a fibrin net is created (Weisel, 2005). Thrombin is part of the relevant cascade that facilitates the conversion (Undas and Ariëns, 2011), and is involved in the normal final step that converts soluble fibrinogen to the resulting fibrin net (Davalos and Akassoglou, 2012). Under laboratory conditions, this fibrin fiber net can be simulated by adding thrombin to either whole blood or plasma (Pretorius et al., 2013c). In healthy individuals, these fibers form a net that lies on top of and around the discoid RBCs. However, in the presence of iron overload, the RBCs are folded and twisted around the fibers, suggesting structural changes to these fibers and/or cells. The fibers are also more compact, and rather than a net, form dense matted deposits known as “atypical parafibrin” (Lipinski and Pretorius, 2013; Pretorius et al., 2013a). When physiological levels of unliganded ferric iron are added to healthy whole blood, the resulting parafibrin becomes resistant to the normal pathways of fibrin degradation (Pretorius and Lipinski, 2013). Also, the RBCs show the same ultrastructure as in iron overload, where they twist around fibers. The structural changes in RBCs in the presence of iron overload, may impact on the rheological properties of the cells. Previously, it was noted in hereditary hemochromatosis, that there is a higher plasma viscosity, resulting in damage to red cells at the level of microcirculation (Ershova et al., 1998). In the light of the compelling evidence that (i) RBCs and fibrin are changed in iron overload, and (ii) that iron is involved in the pathogenesis of AD, we here investigate the hypothesis that in AD, especially in the presence of iron overload, the morphology of RBCs is also altered significantly, and that one may contribute to the other.

Because iron is implicated in AD, serum ferritin (SF) levels of each patient were also measured. Iron deposition in various organs, ultimately resulting in end-organ damage, including arthralgias, osteoporosis, cirrhosis, hepatocellular cancer, cardiomyopathy, dysrhythmia, diabetes mellitus, and hypogonadism (Crownover and Covey, 2013; Kanwar and Kowdley, 2013) has been linked directly to elevated SF levels (Kanwar and Kowdley, 2013). Also, SF above 150 ng/mL^−1^ (females) or 300 ng/mL^−1^ (males) is taken as iron overload (Kellner and Zoller, 1992; Koziol et al., 2001; Adams et al., 2005, 2006; Adams, 2008; Jacobs et al., 2009; Gordeuk et al., 2012; Crownover and Covey, 2013). Light microscopy was used to study the general morphology of the erythrocytes, and their axial ratios were measured to determine variation in shape. Also, scanning electron microscopy (SEM) analysis of RBCs was performed to determine interactions between erythrocytes and fibrin. Furthermore, the presence of spontaneous hypercoagulability, visible as dense matted fibrin deposits (DMDs), and ultrastructural erythrocyte membrane changes were also studied. The deformability of the cell membranes was determined using atomic force microscopy (AFM). Finally, confocal microscopy was used to study the permeability of erythrocyte membranes to a particular dye, to assess the presence of membrane damage. Overall, we found that many AD patients have an increased SF level, and that the RBCs of these individuals differed significantly in both ultrastructural properties and membrane elasticity, as well as in terms of membrane permeability, from those taken from healthy as well as those from AD individuals with normal SF levels.

**Figure 1:**
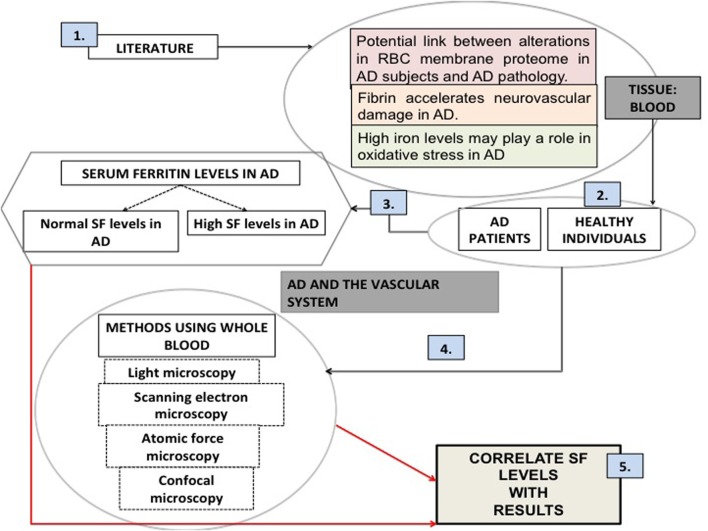
**An overview figure summarizing the contents of this manuscript. (1)** Literature suggests that there is a rationale for looking at the vascular system and particularly blood; **(2)** our sample was chosen to be a random group of Alzheimer's individuals (AD), as well as healthy individuals; **(3)** (unliganded) iron has been implicated in the literature as a role player in AD; serum ferritin levels were measured; **(4)** our methodologies included various microscopy techniques; **(5)** finally we correlated our microscopy results to the presence of high serum ferritin levels.

Thus, in the current work we studied the blood of 25 AD patients and of 40 healthy individuals. This manuscript is structured as follows (see Figure 1, based on the desirability of an overview figure (Wong, 2011): we start with a literature survey of AD and its relationships to the vascular system, particularly the role of RBCs. Then we assessed the SF levels in AD patients. We used four different microscopy techniques to study whole blood from AD patients and compared these to samples taken from healthy individuals. Finally we correlated the microscopy results with SF levels in our AD individuals.

## Materials and methods

### Volunteer details

Bloods were obtained from fully diagnosed Alzheimer's patients. All donors so designated had been diagnosed with AD by qualified medical practitioners and did not smoke. Specifically, a neurologist diagnosed all AD individuals that included the patient history, as well as the Mini Mental State Examination (MMSE) (Table 1 shows their history and SF levels, while Table 2 shows the summary characteristics of the sample. While the average age and gender balance differed between controls and AD patients, they were well matched between low-SF and high-SF AD patients, which is what is of interest here). A recent study suggested that there are increased markers of iron deposition (including SF levels) and oxidative stress in patients with cognitive dysfunction (Umur et al., 2011). Furthermore, the authors suggested that it seems likely that these markers had a negative effect on the MMSE score (Smith et al., 2010; Bartzokis et al., 2011; Umur et al., 2011; Penke et al., 2012). Ethical clearance was obtained from the Health Sciences Ethical committee from the University of Pretoria and informed consent was obtained from family members who act as guardians of the patients. Healthy individuals also filled in consent forms.

**Table 1:** **Serum ferritin levels (SF) of Alzheimer's (AD) patients, history and corresponding figure numbers**.

**AD individual gender and age**	**AD diagnosed in years and previous medical conditions**	**SF (ng·mL^−1^)**	**Corresponding figure**
**AD WITH NORMAL SF**			
F; 74	4; previous thrombotic stroke	49	Figure 3
F; 59	6; epilepsy	58	
F; 91	10; Osteoporosis	32	
F; 85	3; none	17	
F; 67	1.5; high blood pressure	130	Figure 2 (4); Figure 6C (4)
F; 76	13; hypothyroidism	94	Figure 6B (3)
F; 83	3; hypothyroidism	57	Figure 2 (3)
F; 93	13; hypothyroidism; 3 heart attacks	128	Figure 6B (2)
F; 80	12; hypothyroidism; high blood pressure	29	Figure 2 (1)
F; 67	8; high blood pressure; high cholesterol	101	Figure 2 (2); Figure 6B (1)
**AD WITH HIGH SF**			
M; 60	1; high cholesterol	359	
F; 84	10; kidney atrophy	302	Figures 4B,C
F; 96	2; none	256	Figure 2 (3)
F; 77	2; asthma; high blood pressure; osteoporosis	173	Figure 2 (2)
F; 76	7; high cholesterol	194	Figure 6C (2)
M; 74	4; depression	311	Figure 6C (4)
M; 80	15; thrombotic stroke; high blood pressure	187	
F; 85	3; none	189	
F; 93	3; hypothyroidism; heart bypass	240	Figure 2 (4)
F; 88	4; hypothyroidism, high blood pressure; osteoporosis	153	
F; 89	10; high blood pressure	300	Figure 4A
F; 86	9; high blood pressure	244	Figure 6C (3)
F; 78	10; none	423	
F; 75	8; high blood pressure	371	Figure 2 (1); Figure 6C (1)
F; 80	7; back problems	196	

**Table 2:** **Summary characteristics of healthy individuals and Alzheimer's disease individuals**.

	**Healthy individuals (*n* = 40)**	**Normal serum ferritin AD individuals (*n* = 11)**	**High serum ferritin AD individuals (*n* = 14)**
Average age and range	26, 19–79	78, 59–91	81, 60–96
Average number of years suffering from Alzheimer's disease and range	0	8, 2–15	6, 1–10
Male: female	22:18	1:10	2:12
Serum ferritin levels and range	< 150 ng·mL^−1^ for females and 300 ng·mL^−1^ for males	80, 17–187	265, 154–423
Smoker: non-smoker	0:40	0:11	1:13
Etiology ischemic/non-ischemic	0:40	2:9	0:14
Hypertensive: Normotensive	0:40	4:7	5:9
ACE-inhibitors: yes/no	0:40	4:7	3:11
Beta-blockers: yes/no	0:40	1:10	1:13
Hypothyroid: yes/no	0:40	4:7	2:12
Hypercholesterolemia: yes/no	1:39	1:10	2:12
Anti-coagulant: yes/no yes/no	0:40	1:10	1:13
Asthma: yes/no	0:40	0:11	1:13
Epilepsy: yes/no	0:40	1:10	0:14
Osteoporosis: yes/no	0:40	1:10	1:13

*Normal SF females: 10–150 ng·mL^−1^; males: 12–300 ng·mL^−1^; Normal ranges for low density lipoprotein (LDL) < 1.8 mmol·L^−1^; high density lipoprotein: > 1.6 mmol/L; total cholesterol < 5.2 mmol·L^−1^; ischemic vs. non-ischemic: individual had at least 1 thrombotic event in his/her lifetime; non-ischemic: no thrombotic event; normal blood pressure: 80/120 mmHg. Hypothyroidism: thyroid stimulating hormone (TSH) > 5.5 μIU/mL*.

Blood (6 mL) was collected in a citrate tube and also 6 mL were collected in a tube for serum iron level determination. The AD patients were divided into two groups (normal and high SF levels, high “high SF” being defined as a level of >150 ng·mL^−1^ for females and 300 ng·mL^−1^ for males). Healthy individuals were identified and they also did not smoke or have any chronic conditions, their ages varied from 19 to 79. Ethical approval was granted at the University of Pretoria (HUMAN ETHICS COMMITTEE: FACULTY OF HEALTH SCIENCES) under the name J. Bester. Written informed consent was obtained from all healthy individuals used as controls. Written consent was obtained from a family member who is responsible for the care of each AD individual.

### Light microscopy and axial ratios

Thin LM smears were made followed by air-drying, fixing in methanol, and staining with Methylene blue and Eosin (Copenhaver et al., 1971). Slides were mounted with Entellan and viewed with a Nikon Optiphod transmitted light microscope (Nikon Instech Co., Kanagawa, Japan). Axial ratios of RBCs from 40 healthy individuals, as well as the two AD groups were captured using ImageJ (ImageJ is a public domain, Java-based image processing program developed at the National Institutes of Health: http://rsbweb.nih.gov/ij/). Axial ratios were always greater than (or equal to) 1 by using the largest diameter overall as the numerator, and as the denominator the length at 90° to the line used to provide the numerator. On average, 20 RBCs were assessed for each individual. A box plot, which is a descriptive statistical method (and other statistics) were calculated using MS-Excel, together with the add-in template downloadable from http://www.vertex42.com/. *P*-values were calculated from the means, the numbers of objects measured in each class and the standard deviations using the Excel add-in available via http://www.talkstats.com/attachment.php?attachmentid=261&d=1213281245 and the facility at http://www.graphpad.com/quickcalcs/ttest1.cfm?Format=SD.

### Scanning electron microscopy (SEM)

After the blood was collected, 10 μl of whole blood were placed directly on a glass cover slip, fixed, dehydrated, dried, mounted and coated with carbon according to previously described methods (Buys et al., 2013). Additionally, 10 μl of whole blood (WB) was mixed with 5 μl of thrombin, to create an extensive fibrin network in between the RBCs. A Zeiss ULTRA Plus FEG-SEM with InLens capabilities was used to study the surface morphology of erythrocytes, and micrographs were taken at 1 kV.

### Atomic force microscopy (AFM)

WB in a citrate tube was centrifuged at 145× g for 30 s. The supernatant (plasma, platelets, and white blood cells) were discarded and the remaining RBC's were prepared for AFM by fixing in 4% formaldehyde (made up in PBS) for 30 min, at room temperature (22°C) followed by dehydration and placing a drop of the RBC's suspended in undiluted (161.39 g/mol) hexamethyldisilazane (HMDS) onto a glass cover-slip and spreading the fluid by tilting the coverslip sideways, ensuring an even distribution of cells. Cover slips were dried and stored until AFM analysis.

**Figure 2:**
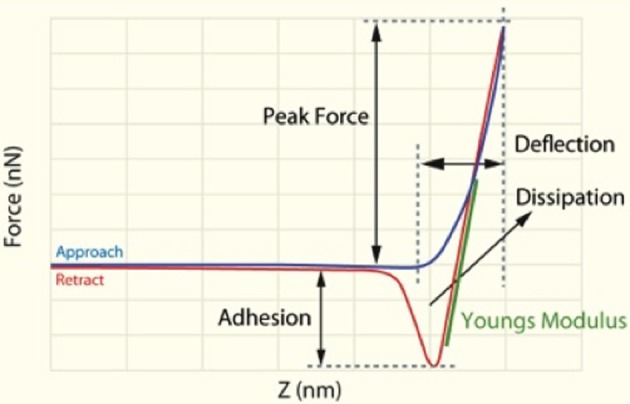
**Schematic representation of force/separation plot illustrating the type of the information that can be obtained [adapted and redrawn from Berquand (2011)]**.

Characterization of the cells was performed with a commercial AFM system (Dimension Icon with ScanAsyst, Bruker, USA) using the PeakForce QNM (Quantitative Nanomechanical Property Mapping) imaging mode. This method is similar to the standard tapping mode of scanning probe microscopy, where the probe and the sample are brought together intermittently, but in contrast to the more classical tapping mode (where the oscillation *amplitude* is kept constant), this mode operates by controlling the *maximum force* applied by the probe to the sample (Dufrêne et al., 2013). At every pixel a rapid force-distance curve is performed and as the cantilever's deflection sensitivity and spring constant is calibrated before measurements, the curve can be analyzed quantitatively to obtain a series of specific property maps of the sample (Figure 2). Thus, the retract curve is used to calculate modulus and adhesion images (slope of the curve and the minimum of the curve, respectively), the variation between the zero and maximum force is used to calculate deformation and the area between the approach and retract curve can be used to calculate energy dissipation (Berquand, 2011; Kolar et al., 2013). Thus, the retract curve is used to calculate modulus and adhesion images (slope of the curve and the minimum of the curve, respectively), the variation between the zero and maximum force is used to calculate deformation, also energy dissipation can be measured as tip-sample interactions cause hysteresis between the approach and retract curves and by measuring the area between these curves the loss of mechanical energy can be determined.

The Young's modulus is a measure of the stiffness of an elastic material and can generally be defined as stress divided by the corresponding strain, with greater values indicating increased stiffness or decreased deformability. As each force curve's data can also be stored individually, it is possible to obtain quantitative measurements of the Young's modulus by fitting the slope of any force distance curve of the image to an appropriate model (in this instance; the Derjaguin–Muller–Toporov (DMT) Model (Derjaguin et al., 1975). Silicon Nitride probes (TAP525—MPP 13120-10, Bruker, USA) with a nominal force constant of 200 N·m^−1^, a resonant frequency between 430 and 516 kHz (measured by the manufacturer), and a nominal tip radius of 15 nm were employed in all AFM measurements. Ten cells from a minimum of 7 individuals out of each group (see Table 3) were analyzed by selecting a 1 μm by 1 μm scan area on the periphery of the RBC and performing 128 by 128 data points of individual force curve measurements with a peak force of 6 μN. The periphery of the cells was chosen as there might be differences in concavity of RBCs, and we therefore chose an area that is not affected by the concavity of the specific RBC. The scans were performed at 0.6 Hz, which translates to a tip velocity of 1.2 μm·s^−1^ and 25–35 force curves were chosen randomly within the stated area. Offline software (NanoScope Analysis version R3, Bruker, USA) was used to process the force curves and fit the modulus model to the unloading portion (red curve, using the green section marked in Figure 2 of the “retraction” curve). The goodness of fit (*R*^2^) between the modulus model and the data given by the acquired curve is determined by calculating the ratio of explained variation to total variation in the dataset; only force curves with a goodness of fit so defined of 0.85 and above were used for modulus measurements. The statistical significance of the difference between calculations was determined using one-way analysis of variance. A *P*-value of less than 0.05 relative to the null hypothesis was considered to be “significant” [cf. (Broadhurst and Kell, 2006)], and *P*-values are noted.

**Table 3:** **Descriptive statistics for elasticity (MPa) of red blood cell (RBC) membranes as obtained from the atomic force microscopy (AFM)**.

**Analysis**	**Control**	**High SF AD**	**Normal SF AD**
Mean	46,711	53,465	45,706
Standard deviation	39,211	49,711	45,473
Standard error	750	1228	788
*P*-value (vs. control)	–	6.6·10^−7^^*^	0.3
*N* (individuals)	11	7	10
*P*-value (high SF AD vs. normal SF AD)		4.4·10^−8^^*^	
*N* (cells)	110	72	100
*N* (curves)	2737	1639	3331

* Significant p-value.

### Confocal microscopy

Confocal microscopy was used to determine if membrane damage could be detected using a specific membrane marker. Unfortunately, there is a limited availablilty of specific markers for RBCs and their membranes. We identified the use of LIVE/DEAD Fixable Dead Cell Stain from Life Technologies™ as a possible marker for membrane damage. This stain kit is based on the reaction of a fluorescent reactive dye with intracellular and extracellular amines. The reactive dye can permeate membranes compromised before fixation and react with free amines both in the interior and on the cell surface, resulting in intense fluorescent staining (Perfetto et al., 2010). In viable cells, only the exterior cell-surface amines are available to react with the dye, resulting in relatively dim staining. The influence of cell type and marker specificity on the stability of fluorescence intensity after fixation has not been well studied and fluorescence stability should be determined for each cell type and marker used (Stewart et al., 2007). Therefore, we optimized the method specifically to use blood fixed and stored in 4% formaldehyde. For this, we used fresh and fixed blood (4% formaldehyde) of three healthy individuals, as well as of an individual with high SF levels diagnosed with hemochromatosis (*SF* = 33000 ng·mL^−1^). Before we added the dye, we washed the fixed samples, 3× with PBS to eliminate formaldehyde residues. No difference in fluorescence intensity was noted between the fixed and fresh samples, except that the fluorescence faded within a minute in the fresh samples.

In the current study, the RBCs were fixed and stored in 4% formaldehyde. When confocal microscopy was done, the cells were washed 3× in PBS and vortexed to form a pellet. 100 μl of RBCs was then re-suspended in 100 μl PBS. 0.2 μl of LIVE/DEAD Fixable Dead Cell Stain (from Life Technologies) was added to 20 μl RBCs in PBS and incubated at room temperature, in the dark for 20 min. 5 μl of the sample were mounted and viewed using a Zeiss LSM 510 META confocal microscope with a Plan-Apochromat 63×/1.4 Oil DIC objective with wavelengths of 488 nm and 514 nm. Confocal micrographs were analyzed using the ImageJ fluorescence measuring function, where we determined the level of fluorescence. We calculated the corrected total cell fluorescence (CTCF) for each patient and then the mean for each of the 3 groups. This is done with the following formula: CTCF = mean integrated density − (mean area of selected cells X mean fluorescence of background readings).

## Results

**Figure 3:**
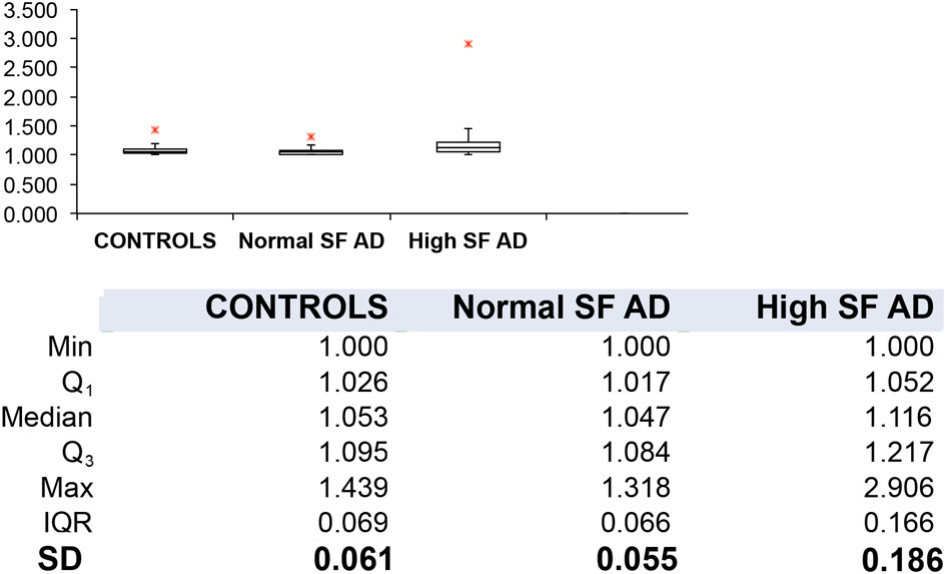
**Box plots and descriptive statistics of the axial ratios of erythrocytes of healthy individuals and of Alzheimer's patients (AD) with normal or high serum ferritin (SF) levels.** There were no significant difference between the axial ratios of RBCs of healthy individuals and those of normal SF AD patients (*p* = 0.099), however, there were significant differences between the axial ratios of erythrocytes from normal SF AD patients vs. those of high SF AD patients (*p* = 1.07·10^−11^) and between those of healthy individuals and those from high SF AD patients (*p* = 1.84·10^−35^).

**Figure 4:**
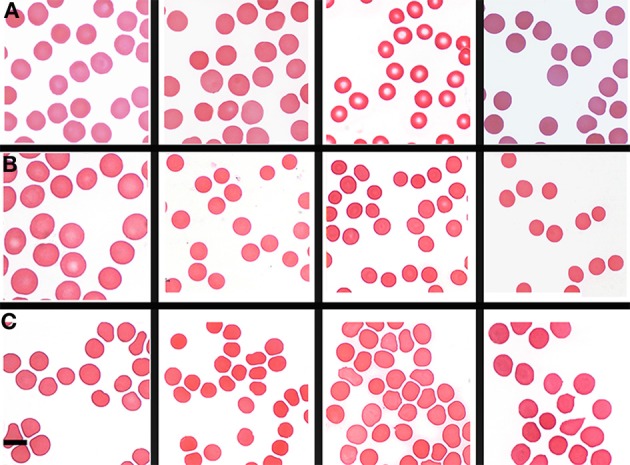
Light microscopy smears of **(A)** four healthy individuals and **(B)** four normal and **(C)** four high normal serum ferritin (SF) Alzheimer's (AD) individuals. Scale = 5 μm.

Table 1 shows the outline medical history of the AD individuals, as well as their SF levels, while Table 2 shows statistical summary characteristics. None of the individuals was considered (as discussed with their neurologist) to have major health complications that might bias the analysis. Figure 3 shows the axial ratios of the erythrocytes, depicted as box plots, together with descriptive statistics. There are no significant shape difference between the erythrocytes of controls and of those taken from AD individuals with normal levels of SF, however, there are highly significant differences between the axial ratios of both control vs. high SF AD individuals and between the axial ratios of the erythrocytes of normal SF and of high SF AD individuals. Figure 4 shows light microscopy smears of four healthy individuals and also four normal and four high SF AD individuals. In these micrographs, healthy individuals show the typical discoid RBC shape. This is also seen in the normal SF AD individuals. However, a large fraction of the RBCs of the high SF AD individuals have an elongated, non-discoid RBC shape, as previously seen for erythrocytes taken from individuals with iron overload (Pretorius and Lipinski, 2013).

**Figure 5:**
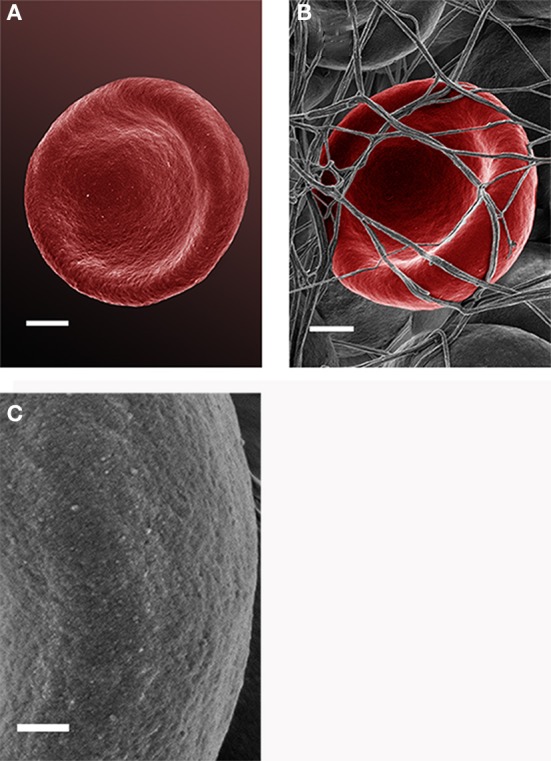
Scanning electron microscopy (SEM) micrographs of a typical healthy red blood cell (scale = 1 μm) **(A)**. RBC that is discoid in shape when thrombin is added to whole blood (scale = 1 μm) **(B)**; high magnification of RBC membrane, showing globular structure; **(C)** (Scale = 100 nm).

**Figure 6:**
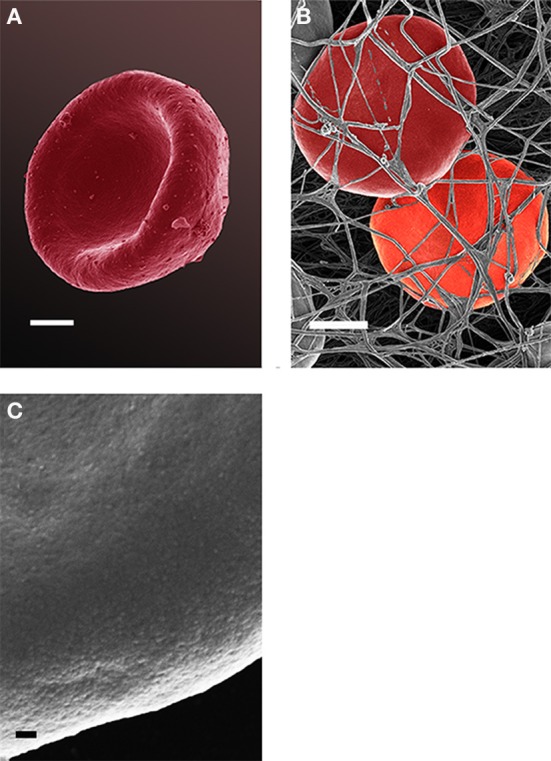
Red blood cell from a normal SF AD individual (scale = 1 μm) **(A)**; where thrombin is added to whole blood; the cells keep their discoid shape (scale = 1 μm) **(B)**; high magnification of RBC membrane, showing globular structure (Scale = 100 nm) **(C)**.

**Figure 7:**
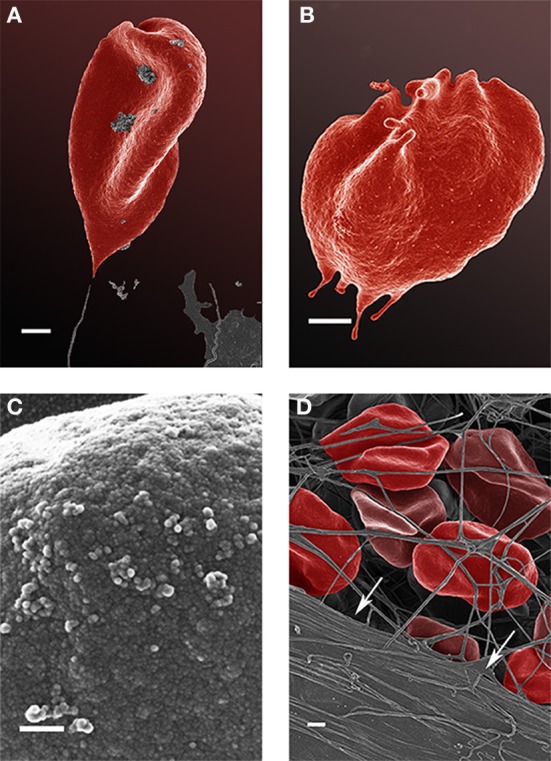
Scanning electron microscopy (SEM) micrographs of a typical high serum ferritin (SF) Alzheimer's (AD) individual (scale = 1 μm) **(A,B)**. High machine magnification (150,000×) of a typical RBC membrane from a high serum ferritin (SF) Alzheimer's (AD) individual (Scale = 100 nm) **(C)**; red blood cell from a high serum ferritin (SF) Alzheimer's (AD) individual where thrombin is added to whole blood; the cells loose their discoid shapes and dense matted fibrin deposits are present in the lower left corner of the micrograph (indicated by arrows) (scale = 1 μm) **(D)**.

Analysis of RBCs using SEM (Figures 5–7) confirmed the LM results, and showed that the RBCs of normal SF AD individuals typically have a discoid shape, and appear no different from the RBCs of healthy individuals. When thrombin is added to whole blood of healthy individuals, the RBCs retain their discoid shape (Figure 5B) (Pretorius, 2013); the same was found for whole blood from the normal SF AD patients (Figure 6B). However, when looking at the RBCs of high SF AD individuals, these were seen to form pointed extensions and to have a slight elongation (in WB smears); this was also noted in LM smears (Figures 4, 6A,B). Also, in the presence of fibrin fibers, generated after addition of thrombin, the RBCs fold around the fibers (Figure 7D), and areas of thickened and matted fibrin or DMDs are also noted (Figure 7D). This changed fibrin ultrastructure, where fibrinogen coagulates to form matted plates rather than individual fibrin fibers (that were also thinner), has previously been noted in both thrombo-embolic ischemic stroke and diabetes (Lipinski et al., 2012b; Buys et al., 2013), as well as in the presence of exogenously added unliganded Fe(III) (Pretorius et al., 2013c).

High (150,000×) SEM magnifications of RBC membranes show that the membrane surface of healthy cells has a typical globular structure (Pretorius, 2013; Pretorius et al., 2013b) (Figure 5C); this was also found for normal SF AD individuals (Figure 6C). The membranes of RBC from high SF AD individuals were more granular, when studied at 150,000× magnification (compare Figures 6C, 7C). This implies that the membrane architecture is changed and that possibly the elasticity and membrane integrity might be compromised. Therefore, because significant changes in membrane ultrastructure were seen when viewed at very high SEM magnification, we were interested to see if these changes were accompanied by any other alterations in the RBC membrane, which may result in changed mechanical properties of the cell.

**Figure 8:**
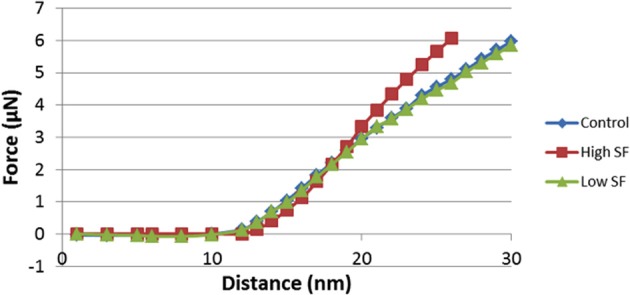
**Force-Distance curves obtained on RBCs from healthy individuals, normal serum ferritin (SF) Alzheimer's (AD) individuals and high serum ferritin (SF) Alzheimer's (AD) individuals.** Force-Distance curves show the atomic force microscope (AFM) cantilever deflection range on the platelet surface.

Force-distance curves [see (Dufrêne et al., 2013) and above], and subsequent modulus value calculations, were used to assess this. Elastic modulus calculations (Table 2) showed that the Young's modulus of RBCs taken from high SF AD individuals (53,465 MPa) is statistically significantly higher than the Young's modulus values observed in control and normal SF AD RBCs (46,711 and 45,706 MPa, respectively). This ca 15% increase in the Young's Modulus, meaning there is a decrease in elasticity, can also be seen directly in the typical force-distance curves (Figure 8), where a steeper slope and decreased probe displacement of the high SF RBC curve is evident.

**Figure 9:**
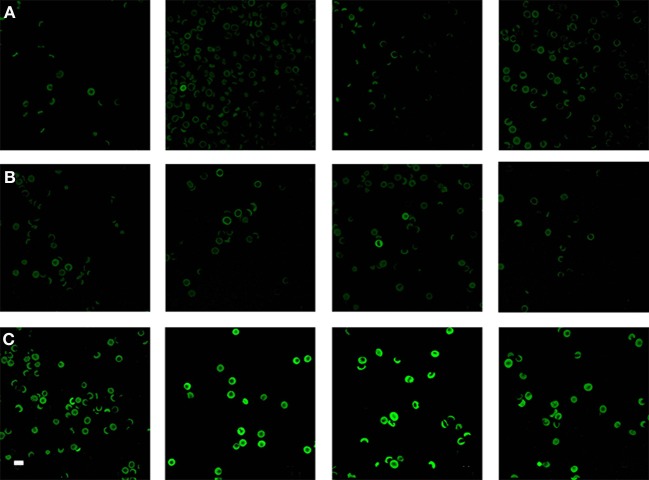
Confocal microscopy of RBCs from **(A)** four healthy individuals, **(B)** four normal serum ferritin (SF) Alzheimer's individuals (AD) and **(C)** four high SF AD individuals. Scale = 10 μm.

Because the changes in mechanical stiffness of the RBCs of AD patients with high SF might reasonably be expected to be reflected in changes in other membrane properties, we then sought to assess the membrane permeability of the different RBC (before fixation) using a so-called LIVE/DEAD Fixable Dead Cell Stain. The LIVE/DEAD Fixable Dead Cell Stain can bind to amines on the outer membrane of the cell but when membrane integrity is compromised it may enter the cell to bind to amines typically found inside the cell. When this happens, the damaged cells will show a higher green fluorescence. To compare confocal microscopy data of RBCs from healthy individuals with those from individuals with AD and normal and high SF, we show micrographs from six individuals in each of the three groups (Figure 9). Confocal microscopy showed that there is substantially higher fluorescence intensity in RBCs from the high SF AD individuals relative to that in RBCs taken from the controls or the normal SF AD individuals. Analysis of confocal micrographs with ImageJ showed the CTCF for each of the groups. A *t*-test was performed at 5% level of significance (i.e., where a *P* = 0.05 is considered as significant). On this basis, there is no significant difference between the CTCF of healthy individuals vs. that of normal SF AD individuals (*p* = 0.89), however, there is a significant difference between the CTCF of healthy individuals and of high SF AD patients (0.04) as well as between normal SF and high SF AD individuals.

**Figure 10:**
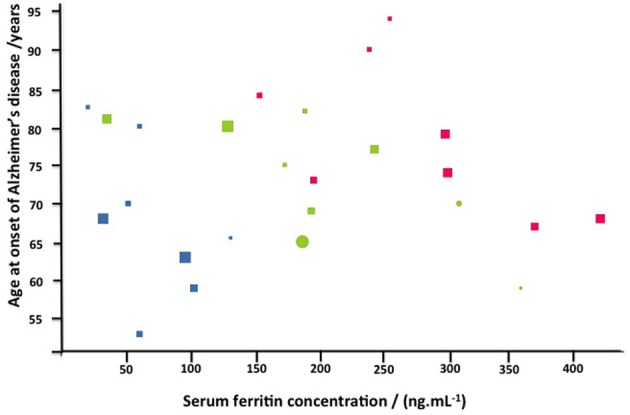
**Alzheimer's disease as a function of serum ferritin for normal (blue) slightly (green) or strongly affected (red) morphologies.** Also displayed are gender (females circles, males squares), and duration in years (via the size of the symbols).

To determine if there is any correlation between the severity of the disease manifestations and other variables, we graded the light microscopy smears independently (Table 4). We used three stages, namely normal discoid shape (**N**, typically seen in healthy individuals), slightly affected (**S**), and severely affected (**A**). There was no especially obvious relationship between the morphological grades and either the age at onset or the duration of the Alzheimer's. However, the SF levels were noticeably greater as the cells moved from grades **N** (67 ± 35 ng·mL^−1^, mean ± *SD*) through **S** (202 ± 91 ng·mL^−1^) to **A** (280 83 ± ng·mL^−1^). Visualizations were also performed using the Tibco/Perkin Elmer Spotfire software, where the trends once again showed that the severity of the RBC changes are clearly linked to the SF levels (Figure 10).

**Table 4:** **Alzheimer's disease patient data, showing duration and age of onset and serum ferritin (SF) levels, as well as severity of changes, indicated as normal discoid morphology, slightly affected and severely affected shape changes**.

**Gender**	**Age at onset of AD**	**Duration in years**	***SF* (ng/mL^−1^)**	**Discoid shape**	**Slightly affected**	**Significantly affected**
F	70	4	49	x		
F	80	3	58	x		
F	82.5	2.5	17	x		
F	65.5	1.5	130	x		
F	63	13	94	x		
F	53	6	58	x		
F	68	12	29	x		
F	59	8	101	x		
M	59	1	360		x	
F	81	10	32		x	
F	75	2	173		x	
F	69	7	194		x	
M	70	4	311		x	
M	65	15	187		x	
F	82	3	189		x	
F	80	13	128		x	
F	77	9	244		x	
F	74	10	302			x
F	94	2	256			x
F	90	3	240			x
F	84	4	153			x
F	79	10	300			x
F	68	10	423			x
F	67	8	371			x
F	73	7	196			x

## Discussion

Iron overload is associated with many pathological conditions, including liver and heart diseases, neurodegenerative disorders, diabetes, hormonal abnormalities immune system abnormalities, heart failure, and in particular in the more classical conditions recognized as “iron overload” diseases such as hereditary hemochromatosis, where iron overload is caused by a specific genetic mutation (Kwan et al., 1998; Shizukuda et al., 2006, 2007; Kell, 2009, 2010; Camaschella and Poggiali, 2011; Crichton et al., 2011; Castellani et al., 2012; Finberg, 2013; Funke et al., 2013; Martines et al., 2013; Pretorius et al., 2013a,c; Tsuchiya and Nitta, 2013). Many workers have suggested that increased iron levels are also associated with cardiovacular events by facilitating oxidative stress, and that iron-induced vascular dysfunction may contribute to the increased incidence of ischemic cardiovascular events (Corti et al., 1997; Horwitz and Rosenthal, 1999; Anderson et al., 2001; Day et al., 2003; Kondur et al., 2009; Zegrean, 2009; Zhao et al., 2010; Meroño et al., 2011; Zacharski et al., 2011; Rajapurkar et al., 2012). Poorly liganded iron is the main culprit and plays a fundamental role in the development of pathology (Kell, 2009, 2010). Moderate iron overload is also known to accelerate thrombus formation after arterial injury, increases vascular oxidative stress, and impairs vasoreactivity (Day et al., 2003). The main kind of mechanism by which iron overload causes tissue damage is considered to be free radical toxicity caused by the excessive levels of the (poorly liganded) metal (Jomova and Valko, 2011; Barton et al., 2012; Ferro et al., 2012; Pra et al., 2012; Crownover and Covey, 2013). Hydroxyl radicals (OH ·) are especially well-known culprits in causing damage to biomolecules, and iron catalyzes the redox-based production of hydroxyl radicals via the Fenton reaction (Goldstein et al., 1993; Wardman and Candeias, 1996; Aisen et al., 2001; Kell, 2009, 2010; Rival et al., 2009; Khechaduri et al., 2013).

Previously, we have shown that RBCs are very sensitive to unliganded iron changes, and that physiological levels of ferric iron, added to WB, cause shape changes in RBCs (Lipinski et al., 2012a; Pretorius and Lipinski, 2013). These shape changes are also present in hereditary hemochromatosis, as well as in cases of hyperferritinemia where the genetic mutation underpinning HH is not present (Pretorius et al., 2013a,b,c, in press). There are four iron measurements that medical practitioners typically will request when iron overload is suspected. They are SF, serum iron, serum transferrin and % transferrin saturation (Brandhagen et al., 2002; Limdi and Crampton, 2004). Serum iron is the amount of circulating iron bound to transferrin; transferrin binds iron and controls the level of free iron in biological fluids. Optimal saturation should be between 20 and 45% [% transferrin binding sites that should be filled (McCullen et al., 2002; Limdi and Crampton, 2004; Daniels, 2010)]. We have indicated previously, that SF levels are the iron-related value that most effectively imply or reflect iron overload (Pretorius et al., 2013a,b,c, in press); and this view is reflected in the literature (Kellner and Zoller, 1992; Koziol et al., 2001; Adams et al., 2005, 2006; Adams, 2008; Jacobs et al., 2009; Gordeuk et al., 2012; Crownover and Covey, 2013)]. In the current study, we used SF levels as an indication of iron overload in our AD patient group. SF levels above approximately females 150 ng/mL^−1^ and males 300 ng/mL^−1^ was taken as implying iron overload (Kellner and Zoller, 1992; Koziol et al., 2001; Adams et al., 2005, 2006; Adams, 2008; Jacobs et al., 2009; Gordeuk et al., 2012; Crownover and Covey, 2013).

Inside cells, ferritin is the major iron storage protein, with 24 subunits forming a cage around up to 4500 iron atoms (Lu et al., 2013). However, the method of ferritin excretion into serum, and what it is that is secreted (just the protein shell subunits, or the entire ferritin molecule) that then degrades liberating iron, remains poorly understood. Ferritin concentrations in serum reflect the iron store of the body (Cook et al., 1974; Hori et al., 2010). Although ferritin itself may protect against oxidative stress by chelating free iron (Torti and Torti, 2002), it can also be a mediator of oxidative stress by releasing free iron (Reif, 1992). Thus, despite its clear usefulness as a clinical tool to assess body iron stores, much of the pathophysiology of SF remains vague. We have, however, come to the conclusion that SF is a good bio-marker, as it is widely used in diagnosing and monitoring iron overload diseases (Adams, 2008; Wang et al., 2010). This would be consistent with a view that the levels of SF do reflect unliganded iron that has been liberated during or following its secretion.

Where high SF levels are present in the AD individuals, and when thrombin is added to WB, the RBCs deform and twist around the resulting fibrin fibers (Figure 6D). In the current study we compared RBCs of AD individuals with normal and high SF levels to RBCs taken from healthy individuals. Interestingly, we found that RBCs from the normal SF AD individuals still keep their discoid shape. Also, when thrombin is added to WB, the cells still appear discoid when entrapped in the resulting fibrin fiber network. However, when we study RBCs from high SF AD individuals, their RBCs have a somewhat more elongated shape. This is seen in LM, as well as SEM micrographs (Figures 2, 3).

We also confirm previous results that suggest that fibrin is altered in AD and it was also shown that fibrin interacts with beta-amyloid protein (Merkle et al., 1996; Choi et al., 2002). Also, literature suggests that fibrinogen can bind iron (Orino, 2013) and that this might be pertinent to the correlation between high ferritin and aberrant fibrin morphology as seen in Figure 7D.

We confirmed that high SF AD RBCs have an increased Young's modulus, and therefore decreased elasticity as seen from the AFM results. This finding is in line with previous research that reported increased Young's modulus values in pathological RBCs (Dulinska et al., 2006) and living cells (Müller et al., 2009). Although the cells in the aforementioned study were only briefly fixed and followed by analysis in fluid, other studies have shown that variances in elasticity of different types of dried Jurkat cells (Cai et al., 2009) and RBCs (Jin et al., 2010; Dufrêne et al., 2013; Picas et al., 2013) can be detected. We suggest that the RBC deformability in AD with high SF levels may be due to membrane and cytoskeletal architectural changes and that this might be the reason why the cells have lost their ability to maintain or return to their discoid shape in the presence of fibrin fibers. Continuous and unobstructed delivery of oxygen to the brain via the RBCs, depends on their membrane fluidity (Tateishi et al., 2001). RBCs are highly deformable and elastic, and this physical property contributes significantly to aiding blood flow in the microcirculation (Baskurt and Meiselman, 2003; Mohandas and Gallagher, 2008). Furthermore, research suggests that blood rheology is altered in various pathophysiological conditions. The extent of deformability may be affected by, amongst others, alterations of the properties and associations of membrane skeletal proteins (Koshino et al., 2012). If RBCs have optimal functioning, their ability to deform improves blood flow in the micro-vessels at high shear rate (Shin et al., 2007), and membrane architecture also plays an important role in the mechanical stability of the cells in the presence of shear forces (Nans et al., 2011). Baines in 2009 suggested that the elasticity of RBCs depends on the dynamic rearrangement of spectrin dimers/tetramers under the shearing forces experienced in circulation (Baines, 2009); and that stable intact helical linker regions are needed to maintain the soft elasticity of spectrin (Mirijanian and Voth, 2008). A decreased deformability is also seen in inflammatory conditions such as diabetes (Shin et al., 2007); a spatial reorganization of the cytoskeleton was also noted in this condition (Starodubtseva et al., 2008). A reduced plasticity in cerebral ischemia (Kowal, 1996) and increase stiffness was also noted in conditions like coronary disease and hypertension (Lekka et al., 2005). It therefore seems as if a decreased deformability and increase in stiffness is common in inflammatory disease, and can be ascribed to a changed elasticity as well as stability. Therefore, the changed elasticity seen in the current study, will impact on the membrane fluidity and deformability of the cell as it moves through the circulation. This may result in atypical tissue perfusion, leading to functional deteriorations, ultimately resulting in disturbed vascular properties (Baskurt and Meiselman, 2003). Confocal microscopy confirmed that RBCs from high SF AD individuals have structural membrane damage, in that the probe assessed enters the cell membrane and binds to the amines inside the cell, causing a bright fluorescence in the cells of this group. The bright fluorescence was present to a much lesser degree in RBCs taken from the normal SF AD individuals or the controls, and here only a dull fluorescence was seen, suggesting that as expected the dye reacts only with to the amines on the outer membrane. Statistical analyses confirmed that there was a significant difference between the CTCF of RBCs from healthy and high SF AD individuals, while there were no significant difference between the CTCF of RBCs from healthy individuals vs. normal SF AD individuals. Grading of the severity of the changes (Table 4 and Figure 10), confirms that the SF levels are well correlated with the severity of the RBC shape changes.

## Conclusion

Despite many years of AD research, we still do not know the best strategies to treat this very debilitating condition, and its likely multifactorial aetiology could be taken to suggest that we need a multi-modal treatment regime [e.g., (Kupershmidt et al., 2012a)]. In the current study we were surprised to find that 60% of our randomly chosen AD population had increased SF levels. Although we have shown in the introductory paragraphs that there is extensive literature suggesting that iron levels have been closely associated with AD pathology and might exacerbate pathology in these patients (see above), iron levels in AD patients are not seen as a compulsory pathology test (personal communications with neurologists treating AD patients), notwithstanding literature indicating the relevance of ferritin dysregulation to AD [e.g., (Connor et al., 1992; Quintana et al., 2006; Friedman et al., 2011; Giambattistelli et al., 2012; De Sole et al., 2013)]. Importantly, our findings support the literature regarding dysregulated iron metabolism, as 60% of our sample shows high SF levels. We show that RBC ultrastructure is changed significantly in the presence of iron overload (as judged by SF). We have demonstrated this with 4 different techniques and statistical analysis confirms this. These changes might impair the oxygen carrying capacity and compromise hemorheology of the RBCs, and additionally cause a strain on the already challenged brain function of these individuals. It seems as if iron overload might be present in AD patients more widely than generally believed. Indeed, iron overload might cause the condition to progress faster than in AD individuals who do not have iron overload, particularly due to the additional hydroxyl radical load. The substantial differences in the RBCs, of high-SF AD patients might suggest that some of the minimally invasive measurements we have performed might be of diagnostic significance, while the potential for chelating or liganding iron might have some therapeutic benefits [as first shown more than 20 years ago (Crapper McLachlan et al., 1991) and more recently (Kupershmidt et al., 2012b)]. We conclude by recommending that AD patients should be screened for iron overload and that, when it is present, a comprehensive treatment regime be implemented to monitor and indeed decrease iron levels throughout the progression of this very debilitating condition.

## Author contributions

Janette Bester Collecting of samples, preparation of samples, SEM, LM, and confocal analysis Antoinette V. Buys AFM analysis Boguslaw Lipinski research idea and manuscript preparation Douglas B. Kell statistical analysis, manuscript preparation. Etheresia Pretorius study leader, SEM and AFM analysis manuscript preparation.

## Ethical clearance and consent

Ethical clearance was obtained from the Health Sciences Ethical committee from the University of Pretoria and informed consent was obtained from family members who act as guardians of the patients. Healthy individuals also filled in consent forms.

### Conflict of interest statement

The authors declare that the research was conducted in the absence of any commercial or financial relationships that could be construed as a potential conflict of interest.
